# Supplementary material for: Rescuing low frequency variants within intra-host viral populations directly from Oxford Nanopore sequencing data
Source: Nat Commun. 2022 Mar 14;13:1321. doi: 10.1038/s41467-022-28852-1 (PMC8921239; doi:10.1038/s41467-022-28852-1)
Supplement: Supplementary file 1 — Supplementary Info [file 41467_2022_28852_MOESM1_ESM.pdf]

Rescuing Low Frequency Variants within Intra-Host Viral Populations directly from Oxford Nanopore sequencing data, Supplementary information

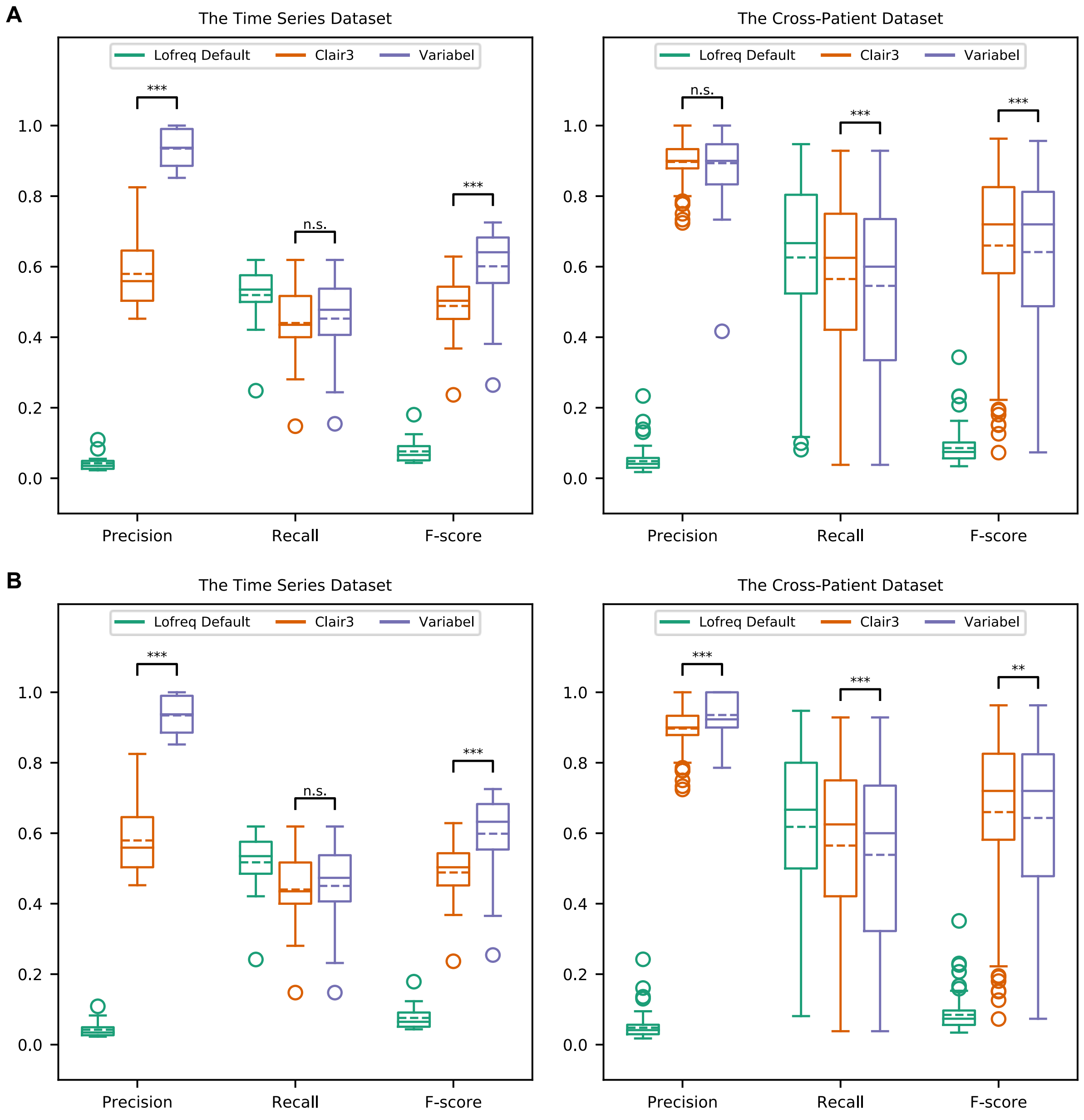

Supplementary Figure 1. Intra-host variant detection on COVID dataset. a) Precision, recall, and f-score comparison of Lofreq default, Clair3, and Variabel on both time series dataset (n=18 samples from the same COVID positive patient collected over distinct time points) and cross-patient dataset (n=103 biologically independent samples collected from COVID positive patients) with minimum coverage set to 10 for Lofreq default and Variabel. Each box plot includes both median line (solid) and mean line (dashed), and the box bounds the interquartile range (IQR). The Tukey-style whiskers extend from the box by at most 1.5 × IQR. The circle denotes outliers that extend beyond the whiskers. Significance between Clair3 and Variabel were calculated using the two-sided paired t-test. Significance labeling:

n.s.(P>0.05), *(P≤0.05), **(P≤0.01), ***(P≤0.001). The exact p-values of the two-sided paired t-test of precision, recall, and f-score between Clair3, and Variabel for the time series dataset are 6.87×10^{-11}, 0.351, and 4.88×10^{-7}. The exact p-values of the two-sided paired t-test of precision, recall, and f-score between Clair3, and Variabel for the cross-patient dataset are 0.631, 2.82×10^{-5}, and 4.05×10^{-4}. **b)** Precision, recall, and f-score comparison of Lofreq default, Clair3, and Variabel on both time series dataset (n=18 samples from the same COVID positive patient collected over distinct time points) and cross-patient dataset (n=103 biologically independent samples collected from COVID positive patients) with minimum coverage set to 50 for Lofreq default and Variabel. Each box plot includes both median line (solid) and mean line (dashed), and the box bounds the interquartile range (IQR). The Tukey-style whiskers extend from the box by at most 1.5 × IQR. The circle denotes outliers that extend beyond the whiskers. Significance between Clair3 and Variabel were calculated using the two-sided paired t-test. Significance labeling: n.s.(P>0.05), *(P≤0.05), **(P≤0.01), ***(P≤0.001). The exact p-values of the two-sided paired t-test of precision, recall, and f-score between Clair3, and Variabel for the time series dataset are 7.24×10^{-11}, 0.451, and 1.25×10^{-6}. The exact p-values of the two-sided paired t-test of precision, recall, and f-score between Clair3, and Variabel for the cross-patient dataset are 9.36×10^{-8}, 6.42×10^{-8}, and 1.95×10^{-3}.

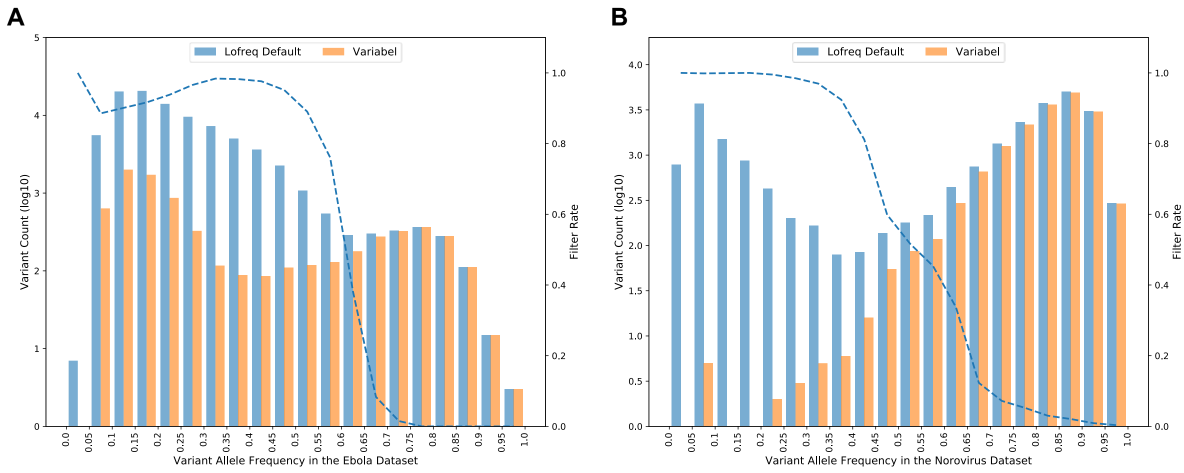

Supplementary Figure 2. Total number of variant calls before and after applying Variabel for the Ebola and the norovirus datasets. a) Histogram with x-axis on the left with log10 scale showing total number of variant calls at each allele frequency range before (blue) and after (orange) applying Variabel for the Ebola dataset (n=118 biologically independent samples collected from Ebola positive patients). The dotted line with x-axis on the right shows the filtering rate of Variabel. **b)** Histogram with x-axis on the left with log10 scale showing total number of variant calls at each allele frequency range before (blue) and after (orange) applying Variabel for the norovirus dataset (n=37 biologically independent samples collected from norovirus GII positive patients). The dotted line with x-axis on the right shows the filtering rate of Variabel.
