# Supplementary material for: Rescuing low frequency variants within intra-host viral populations directly from Oxford Nanopore sequencing data
Source: Nat Commun. 2022 Mar 14;13:1321. doi: 10.1038/s41467-022-28852-1 (PMC8921239; doi:10.1038/s41467-022-28852-1)
Supplement: Supplementary file 3 — Reporting Summary [file 41467_2022_28852_MOESM3_ESM.pdf]

nature portfolio

Corresponding author(s): Todd Treangen

Last updated by author(s): Jan 31, 2022

Reporting Summary

Nature Portfolio wishes to improve the reproducibility of the work that we publish. This form provides structure for consistency and transparency in reporting. For further information on Nature Portfolio policies, see our Editorial Policies and the Editorial Policy Checklist.

Statistics

For all statistical analyses, confirm that the following items are present in the figure legend, table legend, main text, or Methods section.

n/a	Confirmed	
☐	☒	The exact sample size (n) for each experimental group/condition, given as a discrete number and unit of measurement
☐	☒	A statement on whether measurements were taken from distinct samples or whether the same sample was measured repeatedly
☐	☒	The statistical test(s) used AND whether they are one- or two-sided<br>*Only common tests should be described solely by name; describe more complex techniques in the Methods section.*
☒	☐	A description of all covariates tested
☒	☐	A description of any assumptions or corrections, such as tests of normality and adjustment for multiple comparisons
☐	☒	A full description of the statistical parameters including central tendency (e.g. means) or other basic estimates (e.g. regression coefficient) AND variation (e.g. standard deviation) or associated estimates of uncertainty (e.g. confidence intervals)
☐	☒	For null hypothesis testing, the test statistic (e.g. F, t, r) with confidence intervals, effect sizes, degrees of freedom and P value noted<br>*Give P values as exact values whenever suitable.*
☒	☐	For Bayesian analysis, information on the choice of priors and Markov chain Monte Carlo settings
☒	☐	For hierarchical and complex designs, identification of the appropriate level for tests and full reporting of outcomes
☒	☐	Estimates of effect sizes (e.g. Cohen's d, Pearson's r), indicating how they were calculated

Our web collection on statistics for biologists contains articles on many of the points above.

Software and code

Policy information about availability of computer code

Data collection

N/A

Data analysis

The following tools is used in this study:
fastp (v0.20.1)
bwa mem (v0.7.17-r1188)
minimap2 (2.20-r1061)
samtools (v1.11)
lofreq (v2.1.4)
Clair v3
vcftools (v0.1.16)
The code for Variabel is publicly available at: https://gitlab.com/treangenlab/variabel, and we used version 1.0.0 of Variabel for the result and analysis presented in this manuscript.
The code for generating the figures is available at: https://osf.io/qbzgp/?view_only=6cf3b0a15c3e4bfc8529ef725ac660d7

For manuscripts utilizing custom algorithms or software that are central to the research but not yet described in published literature, software must be made available to editors and reviewers. We strongly encourage code deposition in a community repository (e.g. GitHub). See the Nature Portfolio guidelines for submitting code & software for further information.

Data

Policy information about availability of data

All manuscripts must include a data availability statement. This statement should provide the following information, where applicable:

- Accession codes, unique identifiers, or web links for publicly available datasets
- A description of any restrictions on data availability
- For clinical datasets or third party data, please ensure that the statement adheres to our policy

Source data are provided with this paper. The source data, with the manifest of the datasets used in the study, is publicly available in: https://osf.io/7z8xv/?view_only=6cf3b0a15c3e4bfc8529ef725ac660d7. All sequencing data supporting the findings of this study is publicly available.
- The time series COVID dataset: NCBI SRA database under BioProject PRJNA682013 [https://www.ncbi.nlm.nih.gov/bioproject/PRJNA682013].
- The cross patient COVID dataset: NCBI SRA database under BioProject PRJEB41737 [https://www.ncbi.nlm.nih.gov/bioproject/PRJEB41737].
- The Ebola dataset: NCBI SRA database under BioProject PRJEB10571 [https://www.ncbi.nlm.nih.gov/bioproject/PRJEB10571].
- The norovirus dataset: NCBI SRA database under BioProject PRJNA713985 [https://www.ncbi.nlm.nih.gov/bioproject/PRJNA713985].
- The synthetic COVID dataset: China National Center for Bioinformation GSA database with accession number of CRA004499 [https://ngdc.cncb.ac.cn/gsa/browse/CRA004499].

The details about accession numbers of each sequencing run for all datasets used in this study can be found in the manifest.

Field-specific reporting

Please select the one below that is the best fit for your research. If you are not sure, read the appropriate sections before making your selection.

[x] Life sciences [ ] Behavioural & social sciences [ ] Ecological, evolutionary & environmental sciences

For a reference copy of the document with all sections, see nature.com/documents/nr-reporting-summary-flat.pdf

Life sciences study design

All studies must disclose on these points even when the disclosure is negative.

Sample size	No sample size calculation is performed. The sample size for each of the dataset is determined by the size of the datasets that are publicly available. The study uses all samples in one dataset to perform variant calling analysis by searching share variants between samples, and the following sample size if sufficient for our method to identify true positive and false positive variant calls (whether allele frequency of a variant is stable across all samples).<br>Time series dataset: 43 Illumina and ONT sequencing runs in total for a COVID patient.<br>Cross patient dataset: 305 Illumina and ONT sequencing runs in total for COVID patients.<br>The Ebola dataset: 158 ONT sequencing runs for Ebola positive patients.<br>The norovirus dataset: 39 ONT sequencing runs for the norovirus positive patients.<br>Synthetic dataset: 112 ONT sequencing runs for sample of virus-negative nasopharyngeal swab spiked with plasmids containing synthetic S and N genes of SARS-CoV-2 reference genome.
Data exclusions	7 sequencing runs are excluded in time series dataset, and 99 sequencing runs are excluded in cross patient dataset. The runs are excluded because 1) the sequencing runs fails the post-alignment quality control (breadth of genome coverage below 0.9 or average depth of genome coverage below 500), 2) the Illumina/ONT sequencing run can not find matching ONT/Illumina pair or the matching mate fails the post-alignment quality control. After the data exclusion, time series dataset contains 18 pairs of Illumina and ONT sequencing runs, and cross patient dataset has 103 pairs of Illumina and ONT sequencing runs.<br>For the norovirus dataset, 2 ONT samples with breadth of genome coverage less than 0.9 and mean depth of coverage less than 500 are excluded from the study. For the Ebola dataset, since the overall sequencing depth of the samples are low, we lower the minimum mean depth of coverage to 100X but still keep the minimum breadth of genome coverage at 0.9, and 40 ONT sequencing runs are excluded.
Replication	All the experiments are replicated at least twice. All attempts at replication were successful.
Randomization	There is no randomization in this study since<br>1) Sequencing runs are paired, same biological sample are being sequenced twice with Illumina and ONT sequencing platform. The Illumina data was used as benchmark to validate the accuracy of the ONT variant calling results. The data is allocated based on different sequencing platforms, and covariates is not relevant to the case since the data of different groups is generated with the same sample.<br>2) For non-paired samples, the entire dataset is used to evaluated the performance of Variabel against results before applying Variabel, and data is allocated in a single group.
Blinding	Researchers are not blinded during data collection and analysis, since the software make variant calls by comparing samples and all ONT sequencing samples in one dataset are used as inputs for the software to run.

Reporting for specific materials, systems and methods

We require information from authors about some types of materials, experimental systems and methods used in many studies. Here, indicate whether each material, system or method listed is relevant to your study. If you are not sure if a list item applies to your research, read the appropriate section before selecting a response.

Materials & experimental systems

n/a	Involved in the study
☒	☐ Antibodies
☒	☐ Eukaryotic cell lines
☒	☐ Palaeontology and archaeology
☒	☐ Animals and other organisms
☒	☐ Human research participants
☒	☐ Clinical data
☒	☐ Dual use research of concern

Methods

n/a	Involved in the study
☒	☐ ChIP-seq
☒	☐ Flow cytometry
☒	☐ MRI-based neuroimaging
